# Washington State Dental Society

**Published:** 1892-08

**Authors:** 


### EDITORIAL.

### Washington State Dental Society.

The fifth annual meeting of the Washington State Dental Society was held at Seattle, May 5th, 6th and 7th, 1892. Quite a large number were in attendance and much good work done. An interesting programme had been prepared and circulated to the profession. The following officers were elected for the ensuing year: President, Geo. B. Hayes, of Tacoma; First Vice-President, W. E. Burkhart, of Tacoma; Second Vice-President, P. A. Purdy, of Seattle; Secretary, F. P. Hicks, of Tacoma; Treasurer, B. S. Scott, of Ellensburgh. The next meeting will be held at Olympia, May, 1893. A special meeting will be called in the autumn to consider and take action, if need be, upon an amendment to the present State dental law.

The organization of dental societies in the new States of our Union speaks well for the future of the profession. Nearly all the States and territories now have such organizations, and we trust the remaining few will not be slow to organize and have their State societies.

THE profession of Tacoma has its city dental society which has been in operation for three of four years, and is doing most excellent work. In this body the utmost good-fellowship prevails and much is being done for the progress of the profession in that city by this means. The regular meetings are held monthly in rooms arranged expressly for their use. A very creditable museum has been secured, and especially so for so young a society. This is an example to many older and larger societies which they would do well to follow. We would suggest to the members, however, that they do not rest satisfied simply with building up a good museum, but secure a library as well; a good library for a dental society is equally if not more valuable than a museum. Long live Tacoma Dental Society.

